# From “toads” to “princes”—a hermeneutic study of the Chinese translation of *Counselling for Toads: A Psychological Adventure*

**DOI:** 10.3389/fpsyg.2025.1520187

**Published:** 2025-03-04

**Authors:** Huiping Wang

**Affiliations:** Shanghai Normal University, Shanghai, China

**Keywords:** psychotherapy, hermeneutics, fusion of horizons, translation, transactional analysis

## Abstract

The translation of psychotherapy texts has received increased attention in recent years. However, there is still insufficient study on the translation of psychotherapy guidebooks. This study aims to investigate the translation of psychotherapy guidebooks under the guidance of Gadamer’s theory of philosophical hermeneutics through a case study of the Chinese translation of *Counselling for Toads: A Psychological Adventure*. By analyzing two Chinese versions of the book, the study demonstrates how efficient translation can promote the fusion of horizons between the author, the translator, and the target reader. The findings suggest that the translator of psychotherapy guidebooks should first endeavour to expand their horizon by accumulating psychotherapy knowledge to avoid incorrect or insufficient understanding. Then the translator should endeavour to engage in a dialogue with the author/ST and grasp the essence of the key concepts by referring to the entire knowledge structure of psychotherapy. Finally, the translator should strive to render the text as they interpret it in a flexible manner to ensure that the target reader, who may lack psychotherapy knowledge, can fully understand the translated text.

## 1 Introduction

*Counselling for Toads: A Psychological Adventure* was written in 1997 by British psychotherapist Robert de Board. Interestingly, Board chose Mr. Toad from *The Wind in the Willows*, a popular work of 20th-century children’s literature, as the book’s protagonist. In this book, Toad receives therapy from a counsellor named Heron over the course of ten sessions. Following the sessions, Toad, who initially experiences severe depression, finally releases his burdens and marches joyously into the future (Board, 1997). Although the book features several characters from *The Wind in the Willows* and appears to be written for children, it is actually a serious book that serves as an introduction to psychotherapy for general readers. As West pointed out:

The book has some of the trappings of a novel, but it would be unfair to judge it as a work of literature. The author, who himself is a psychotherapist, is much more concerned with demystifying the experience of undergoing counselling than with creating a true sequel to Kenneth Grahame’s *The Wind in the Willow*s (West, 2000, p. 323–324).

In 2012, this book was first translated into Chinese by Zhang Meihui, a professional literary translator, and published in China. Later, Zhang’s translation was revised and republished several times. However, this book did not cause a stir until another Chinese version translated by psychotherapist and English lecturer Chen Ying came out in 2020 and became a bestseller with a total circulation of over 3 million copies.

Eric Berne, the founder of Transactional Analysis (TA), once said, “We’re all born princes, and the civilising process turns us into frogs” (Board, 2013, p. 4). The author deliberately chose Mr. Toad as the protagonist because toads, like frogs, represent the power of change. As children, we cannot change the way our parents treat us, but as adults, we can make a decision about whether we want to live the life that our parents impose on us and what, if any, changes we wish to make. The appeal of the book *Counselling for Toads: A Psychological Adventure* lies in that it presents the process during TA therapy sessions in an understandable and engaging way, so that general readers without prior knowledge of psychotherapy can get a sense of what TA is and experience the process of change along with Mr. Toad.

This paper will discuss the translation of psychotherapy guidebooks with the case study of the Chinese translation of *Counselling for Toads: A Psychological Adventure*, based on Gadamer’s theory of hermeneutics in an attempt to explore an efficient approach to translating books of this special type. This paper is composed of six sections. Section 1 is introduction; section 2 is literature review; section 3 describes the research objectives, materials and methodology; section 4 presents a comparative case analysis by discussing specific translation examples; section 5 provides a discussion on the results of the case analysis; section 6 is conclusion.

## 2 Literature review

### 2.1 Translation of psychotherapy texts

Recently, there has been increased interest in the translation of psychotherapy texts. Many researchers have explored the translation and cross-cultural adaptation of clinical psychotherapy materials, such as questionnaires or instruments, and have suggested the need to modify the original text appropriately for the cultural context of the target language (McGreevy et al., 2014; Rosa et al., 2018; Paiano et al., 2019). Another major approach is addressing the technical problems of translation encountered by interpreters in bilingual/multilingual psychotherapy settings (Bradford and Abilio, 1993; Rechtman, 1997; Milne, 2017). The findings suggested the importance of direct or literal translation in a psychotherapy session that involves different languages and cultures, although there might be exceptions to this rule, such as with translating figures of speech (Bradford and Abilio, 1993). Moreover, some studies have investigated the translation techniques used in academic psychotherapy books. For example, Corcodel and Gabriela (2022) studied the terminology translation of the book *TA Today: A New Introduction to Transactional Analysis* from English to Romanian, listing various direct and indirect translation techniques. They recommended that translators should go beyond correspondences at the level of individual terms and be able to establish interlinguistic references to entire knowledge structures. Weiss (2024) commented on Solms’ *Revised Standard Edition of the Complete Psychological Works of Sigmund Freud* and discussed the challenges of tranlslating Freud’s language. He argued that translation is an ongoing process that carries forward and explores different layers of meaning. Additionally, with the popularization of psychotherapy, it is important to note that there is a growing body of literature that investigates strategies for translating psychotherapy guidebooks, that is, books which introduce psychotherapy to general readers (Wang, 2020; Ren, 2021; Dong, 2022). However, most of these studies are theses by postgraduates based on their own translation practises, and despite having a variety of theoretical approaches, the translation quality still needs improvement in general.

Overall, the translation of psychotherapy texts is a challenging task that demands a high level of competence and knowledge. While some research has been carried out on the translation of psychotherapy guidebooks, further exploration and development of efficient practises are in urgent need in this field.

### 2.2 Gadamer’s theory of philosophical hermeneutics

Hermeneutics is a philosophical discipline concerned with understanding and interpreting texts. The history of hermeneutics can be traced back to ancient Greece. Since the 18th century, hermeneutics has become a more systematic discipline. In the 20th century, the German philosopher Hans-Georg Gadamer constructed the system of philosophical hermeneutics. One of his most significant concepts is *fusion of horizons*. Gadamer asserted that all understanding and interpretation of texts is situated within a particular horizon of historical, cultural, and linguistic contexts. Gadamer (2004) defined “horizon” as “the range of vision that includes everything that can be seen from a particular vantage point.” Gadamer (2004, p. 305) believed that “understanding is always the fusion of these horizons supposedly existing by themselves.” When the interpreter’s horizon intersects with the horizon of the text, the fusion of horizons takes place resulting in a new understanding of that text. A closely-related concept is “prejudice,” developed from Heidergger’s “pre-understanding,” which Gadamer takes as the condition of understanding. Gadamer (2004, p. 273) pointed out that “‘prejudice’ means a judgement that is rendered before all the elements that determine a situation have been finally examined.” Another major concept of Gadamer is *Hermeneutical Circle*, which implies the meaning of a text is constructed through an ongoing conversation between the interpreter and the text. Gadamer (2004, p. 189) asserted that “Understanding is always a movement in this kind of circle, which is why the repeated return from the whole to the parts, and vice versa, is essential.” Therefore, “we must understand the whole in terms of the detail and the detail in terms of the whole” (2004, p. 291).

Gadamer’s hermeneutic theory has profound implications for translation. Firstly, it suggests that translation should go beyond the mere transmission of words from one language to another. To achieve the fusion of horizons, the translator should establish the dialogical interaction with the text and the target reader and forge a new horizon. Gadamer (2004, p. 386) asserted that “understanding does not really take place between the partners of the conversation, but between the interpreters, who can really have an encounter in a common world of understanding.” Secondly, a degree of creativity and flexibility in translation is required. Gadamer (2004, p. 388) pointed out: “Translation, like all interpretation, is a highlighting. A translator must understand that highlighting is part of his task. Obviously he must not leave open whatever is not clear to him. He must show his colors.” He claimed that “every translation is at the same time an interpretation. We can even say that the translation is the culmination of the interpretation that the translator has made of the words given him” (2004, p. 386). According to Gadamer (2004, p. 387), translation is “a re-creation of the text guided by the way the translator understands what it says.”

Enlightened by Gadamer’s theory, extensive research has been conducted in the field of translation. The majority of them involve the translation of literary works such as poetry (e.g., Ding, 2012; Zhong, 2018; Sarvghadi and Taebi, 2021), novels (e.g., Liu and Ren, 2010; Wu et al., 2020; Peng, 2021; Li, 2021), as well as ancient classics (e.g., Tan, 2012; Xu, 2014; Dai and Gu, 2019; Yan, 2020; Li and Sun, 2021). The findings of these studies have manifested the importance of achieving the fusion of horizons in translation via different case studies. So far, however, there has been very little discussion on the translation of psychotherapy texts through the lens of Gadamer’s theory of hermeneutics.

## 3 Research objectives, materials and methodology

Following the previous review, this study aims to explore the translation of psychotherapy guidebooks under the guidance of Gadamer’s theory of philosophical hermeneutics. This study seeks to demonstrate the effectiveness of this approach in facilitating understanding and to outline an appropriate strategy for translating books of this special type to provide a reference for future translators.

To achieve these objectives, a comparative case analysis will be conducted on the Chinese translation of the psychotherapy guidebook *Counselling for Toads: A Psychological Adventure*. Two Chinese versions of the book will be examined, which are *蛤蟆先生的希望* “Hope of Mr. Toad” (Board, 2013) translated by Zhang and published in 2013 by Yilin Press (TT1), and *蛤蟆先生去看心理医生 “*Mr. Toad Going to See the Psychotherapist” (Board, 2020) translated by Chen and published in 2020 by Tianjin People’s Press (TT2). These two translated texts were collected following the principles of representativeness and comparability. First, the two translators have different identities: Zhang is a professional Chinese translator mainly engaged in the translation of literary works, whereas Chen is a Chinese psychotherapist and English lecturer who has rich experience in counselling for patients. Second, the two versions differ in translation purposes. TT1 was categorised as children’s literature when published, whereas TT2 was categorised as psychotherapy guidebook, which correspondingly exerted influence on their translation approaches.

The comparative case analysis will cover three aspects: analyzing the source text (ST) to identify key concepts and terms related to psychotherapy; comparing the translated texts with the ST to identify any discrepancies in meaning; and comparing the two translated texts to identify differences in translation approaches. An in-depth discussion utilising Gadamer’s theory of philosophical hermeneutics will follow to explore the reasons behind the popularity of Chen’s translation and how efficient translation can promote the fusion of horizons between the author, the translator, and the target reader.

## 4 Analysis

The author of *Counselling for Toads: A Psychological Adventure* creatively integrates three elements in his book-classic children’s literature, the theory of psychotherapy TA, and the delicate counselling process, in an attempt to introduce the complex process of counselling to a general reader (Board, 2013, p. 1). TA is a psychological theory developed by Eric Berne in the 1950s, and is based on four foundational pillars:

- The structural model of ego states-the structure of personality.
- Transactions-which deal with communications between people.
- Psychological games-repetitive sequences of transactions.
- Scripts-life patterns (Tudor, 2002, p. 20).

According to Berne, a transaction can be defined as:

…consisting of a single stimulus and a single response, verbal or non verbal [as] the unit of social action. It is called a transaction because each party gains something from it, and that is why he engages in it. It is the analysis of transactions as a basic social unit of communication in terms of ego states which forms the basis of this theory of social action and interaction (Berne, 1975, p. 20–21).

Stewart suggested that the counselling process involves “a continual three-way interplay between contract, diagnosis and treatment direction” (Stewart, 1996, p. 15), which can be represented as a treatment triangle. The following case analysis will be conducted in accordance with the three phases of the counselling process: contract, diagnosis and treatment, with an emphasis on such key concepts of TA as structural analysis, game, script, and decontamination, to expound the different approaches adopted by the two translated texts when replicating the process of counselling as well as the different effects on the target reader. Firstly, the phase of contract involves a mutual commitment by both parties in counselling, as Tudor (2002) highlights. Secondly, the phase of diagnosis emcompasses three fundamental concepts: structural analysis, game, and script. Berne (1961) describes “structural analysis” as being concerned with the segregation and analysis of ego states, whereas a “game” is characterised as a pattern of behaviour in which people engage in order to fulfil their needs for attention, recognition, validation, and other psychological rewards. Berne (1975, p. 418) defines a “script” as “an ongoing program, developed in early childhood under parental influence, which directs the individual’s behaviour in the most important aspects of his life.” Finally, the phase of treatment introduces the pivotal concept of “decontamination,” which Tudor (2002) explains as the process of purifying or cleansing an ego state that has been contaminated.

### 4.1 Contract

One of the philosophical tenets of TA is that everyone has the capacity to think. In the counselling contract is mutual, that is to say both parties take responsibility for the procedure; it contains the client’s explicit intention for change (Tudor, 2002, p. 29). In translation, it is crucial for the target reader to realise the importance of the contract at the beginning. See Example 1:

**Example 1**

**ST**: “That is a very good question and I will answer it.” the Counsellor responded. *“Counselling is always a voluntary process, both for the counsellor and for the client*…”

**TT1**: “这个问题很好, 我来回答。*咨询必须是自发的, 对咨询师和当事人都是如此。……*”[Counselling should be voluntary, both for the counsellor and for the client.]

**TT2**: “这是个非常好的问题, 我来回答你。“咨询师回应道, “*心理咨询向来是一个自发的过程, 咨询师和来访者都得出于自愿。……*” [Counselling is always a voluntary process, so both the counsellor and the client should act of their own will.]

Toad did not know at first why he should go to counselling and only felt obliged to do so because of the urging of his friends. At their first meeting, Heron clearly pointed out why Toad should go for counselling-of his own will and for his own benefit. TT2 retains the words “always” (向来) and “process” (过程) from ST, which are omitted in TT1. In addition, TT2 has a more emphatic tone than TT1 by adding the words “都得出于自愿” (should act of their own will), thus emphasising the importance of a contract. Obviously, TT2 has more accurately captured the first essential step in a TA therapy, thus helping to establish the contract between the patient and the therapist in the translated text. Should the target reader be aware of the importance of the contract, they would be more willing to engage in the counselling process together with Toad.

### 4.2 Diagnosis

#### 4.2.1 Structural analysis

As mentioned earlier, structural analysis is concerned with the segregation and analysis of ego states. TA is based on the idea that individuals have three ego states-Parent, Adult, and Child. A Parental ego state is a set of feelings, attitudes, and behaviour patterns which resemble those of a parental figure. The Adult ego state is characterised by an autonomous set of feelings, attitudes, and behaviour patterns which are adapted to the current reality. The Child ego state is a set of feelings, attitudes and behaviour patterns which are relics of the individual’s own childhood (Berne, 1961).

Moreover, the Child is exhibited in one of two forms. The adapted Child is manifested by behaviour which is inferentially under the dominance the Parental influence, such as compliance or withdrawal. The natural Child is manifested by autonomous forms of behaviour such as rebelliousness or self-indulgence (Berne, 1961, p. 77). It is the Parental influence which determines whether the adapted Child or the natural Child is active at a given moment (Berne, 1961, p. 34). See Example 2:

**Example 2**

**ST**: “I do not quite understand,” said Heron. “Why should that make you feel unhappy?” “Because of his attitude,” replied Toad. “Naturally, I was dying to tell him all about my adventures and I began to recount them even before my clothes were dry. *But instead of being interested, Rat accused me of ‘swaggering’*…”

**TT1**: “我不太明白, 这为什么会让你不开心?”“是因为他的态度。我当然急着告诉他我的冒险, 我甚至衣服还没干就开始说了。*但他不但没兴趣, 还指责我‘吹牛’*……” [But he (Rat) not only did not show any interest, but also accused me of “swaggering.”]

**TT2**: 我不太明白, “苍鹭问, “为什么这会让你感到不快乐?”“因为他对我的态度。“蛤蟆答道, “当然, 我很想立刻告诉他我所经历的一切, 还没等衣服干, 就开始跟他讲我的历险。*可他非但没兴趣听, 还说我吹牛, 指责我‘寒碜、邋遢、丢人现眼’*……”[But he (Rat) not only did not show any interest of listening, but also accused me of “swaggering,” and blamed me for being “shabby, slovenly, and disgraceful.”]

Berne pointed out that vocabulary is one of the diagnostic criteria for the therapist which demonstrate the characteristics of an active ego state, and listed different vocabulary corresponding to each ego state:

Typical Parental words are: cute, sonny, naughty, low, vulgar, disgusting, ridiculous, and many of their synonyms. Adult words are unconstructive, apt, parsimonious, desirable. Oaths, expletives, and epithets are usually manifestations of the Child (Berne, 1961, p. 73).

In Example 2, Heron is trying to help Toad find out why he feels so unhappy by making him recall past events in his life. The word “swaggering” used by his friend, Rat, suggests that Rat is demonstrating his Parental ego state in front of Toad, which exerts a negative influence on him. To better illustrate Rat’s Parental influence, TT2 inserts more words with parental characteristics into the translation-寒碜、邋遢、丢人现眼 “shabby, slovenly, and disgraceful,” which leave a deeper impression on the target reader and better illustrate the cause of Toad’s unhappiness. Here is another example:

**Example 3**

**ST**: “The Adult Ego State,” replied Heron, “is the *rational, unemotional*^①^ way we have of behaving. It enables us to *deal with the reality of what is happening in the here-and-now*^②^.”

**TT1**: “成人自我状态是*理性、镇定的*^①^行为表现, 让我们能*处理当下的真实状况*^②^。” [rational and calm^①^; deal with the real situation at present^②^].

**TT2**: “‘成人自我状态’, 指我们用*理性而不是情绪化的*^①^方式来行事。它让我们能*应对此时此地正在发生的现实状况*^②^*。*”[rational rather than emotional^①^; coming to terms with the current situation, which is happening in this moment and at this place^②^].

In Example 3, Heron explains to Toad the meaning of the Adult ego state. TT2 translates “rational, unemotional” from ST as 理性而不是情绪化的 “rational rather than emotional,” while TT1 translates it as 理性、镇定的 “rational and calm.” In comparison with TT1, TT2 better highlights the “rational” feature of the Adult ego state by contrasting it with “emotional.” Moreover, “deal with the reality of what is happening in the here-and-now” from ST is replaced by TT2 with the translation 应对此时此地正在发生的现实状况 “coming to terms with the current situation, which is happening in this moment and at this place.” This translation employs amplification, a technique that expands upon the information provided in the original text. This approach not only enhances the forcefulness of the translation compared to TT1 but also facilitates a deeper understanding for the target readers of the reality-testing ability of the Adult ego state, which are crucial for managing stressful situations.

#### 4.2.2 Game

As mentioned earlier, a game refers to a pattern of behaviour. It usually follows a series of complementary transactions which is broken by one party, perhaps out of an unconscious resentment, who then crosses the communication, with resulting confusion and “bad” feelings on both sides (Berne, 1961, p. 23). See Example 4:

**Example 4**

**ST**: “I suggest that you are,” answered Heron. “*You play a very good game of PLOM*.^①^” “Plom? What on earth is that?” asked Toad. “*PLOM stands for “Poor Little Old Me*”^②^ and you win it every time...”

**TT1**: “我认为你有, *你很成功地玩了一手PLOM的游戏*。^①^”“PLOM?那到底是什么?”“*就是“可怜的我*”^②^, 你每次都成功。……” [You succeed in playing a game called PLOM^①^; That is “poor me.”^②^].

**TT2**: “我认为你在玩游戏, ” 苍鹭答道, “*你很会玩一个叫‘PLOM’的游戏*。^①^”“PLOM?这到底是什么呀?” 蛤蟆问。“*PLOM代表了四个英文单词, 意思就是‘可怜弱小的我*’^②^呀。这个游戏你每局都赢了……” [You are adept at playing a game called PLOM^①^; PLOM stands for four English words, which mean “poor, weak, little me.”^②^].

Toad’s depression is partly due to his unsuccessful transactions with others. He tends to feel sorry for himself and become frustrated, which Heron refers to as PLOM. Heron pointed out his problem to Toad by saying, “You play a very good game of PLOM.” TT1 translates this as 你很成功地玩了一手PLOM的游戏 “You succeed in playing a game called PLOM” and TT2 as 你很会玩一个叫‘PLOM’的游戏 “You are adept at playing a game called PLOM.” Actually, “play a very good game” from ST does not mean that Toad plays the game successfully, but rather that he is inclined to do so, therefore TT2 conveys the contextual meaning more accurately. In addition, for the acronym PLOM, TT2 provides a more detailed explanation than TT1 and helps the target reader to fully understand the implication of the game played by Toad.

#### 4.2.3 Script

The script refers to a set of unconscious decisions a person makes based on their early life experiences that determine their worldview and behaviour patterns. As Stewart and Joines (1987) pointed out, the script is a specific life plan and is decisional and redecisional.

There is a close relationship between the script and life position. The life position an individual takes shapes how they respond to others and the situations they encounter. A positive life position, that is, I’m OK-You’re OK, leads to healthy relationships and positive outcomes, while negative life positions can lead to negative outcomes. In translation, it is important to make the target reader understand the influence the life position exerts on one’s entire life. See Example 5:

**Example 5**

**ST**: “My dear Toad,” said the Heron patiently, “the whole point is that these are *life positions*. Once we decide on these attitudes as children, *we hold on to them for the rest of our lives.* ^②^…”

**TT1**: 苍鹭耐心地说:“亲爱的蛤蟆, 重点就在于这些是攸关一生的*心理地位*^①^。一旦我们在童年决定了何种态度, *就会一辈子抓着不放。*^②^……”[psychological status^①^;(we) will hold on to them for the rest of our lives.^②^].

**TT2**: “亲爱的蛤蟆, ” 苍鹭耐心地回答, “一切的关键就在于那是*“人生坐标”*。^①^一旦我们在童年决定用哪种态度和观点, *我们就会在随后的人生里始终坚持自己的选择。*^②^……”[life coordinates^①^; we will always stick to our decisions for the rest of our lives.^②^].

In Example 5, TT1 translates “life positions” as 心理地位 “psychological status,” while TT2 uses a metaphor and translates it as 人生坐标 “life coordinates.” In comparison, TT2 shows the three-dimensional life positions more vividly and highlights their influence on one’s entire life. For the part “we will hold on to them for the rest of our lives,” TT1 uses literal translation, while TT2 amplifies by translating it as 我们就会在随后的人生里始终坚持自己的选择 “we will always stick to our decisions for the rest of our lives.” In this way, TT2 emphasises that the script is decisional in nature, helping the target reader understand that it is the decisions we make that influence the rest of our lives.

### 4.3 Treatment

A key aspect of TA treatment is help to decontaminate the Adult ego state. According to TA, contamination refers to the transfer of negative feelings and emotions from one ego state to another. Decontamination, on the other hand, refers to the process of purifying or cleansing an ego state that has been contaminated. The result of decontamination is to come into contact with repressed feelings. The more that these feelings are accepted as being OK, the more the symptoms which have been a replacement for those feelings can disappear. Finally, the therapeutic aim is to enable the client to behave in and from their Adult ego state so that the Adult can maintain control of the personality in stressful situations (Tudor, 2002). See Example 6:

**Example 6**

**ST**: *He had not just been rude to Heron. He had taken him on and in some way, overthrown him*, and somehow this had also involved his father. Suddenly he felt that he no longer needed to play the subservient role. *He could assert himself and say what he wanted to say.*

**TT1**: *他不只是对苍鹭无礼, 而是在和他对抗。从某种角度来看, 甚至可以说推翻了他*^①^, 而这似乎也和他的父亲有关。突然间他发现自己不必再扮演卑躬屈膝的角色了；*他可以大声表达自己的意见, 说他想说的话*。^②^[He (Toad) had not just been rude to Heron, but fighting against him. In some way, he had overthrown him^①^; He could speak out his opinion aloud and say what he wanted to say. ^②^].

**TT2**: *他不仅仅是言辞粗鲁, 还顶撞了苍鹭, 从某种程度上说, 是把苍鹭打倒在地*^①^, 而这不知怎么又和他父亲有关系。他突然感到再也不用扮演那个卑躬屈膝的角色了, *他可以说自己想说的话, 还能说得掷地有声*。^②^[He (Toad) had not just been rude to Heron in his words, but contradicted him defiantly. In some way, he had knocked Heron down to the ground^①^; He could speak out what he wanted to say, in a forceful and lofty manner.^②^].

After several sessions of counselling, Toad finally had the courage to come into contact with his repressed feelings. He gave free rein to his anger at Heron, which is symbolic of his overthrowing his father. Compared to TT1, TT2 adopts more descriptive vocabulary and a stronger tone, such as 顶撞 “contradict defiantly,” 打倒在地 “knock down to the ground,” and 掷地有声 “in a forceful and lofty manner.” These vivid descriptions indicate the process of decontamination of Toad’s Adult ego state: he is no longer the submissive child who bows to his father’s parental influence, but has transformed into an adult who is fully in control of himself and able to make rational decisions. Thus, the target reader can get a clear picture of how Toad develops from the adapted Child ego state to the Adult ego state, hence completing the change from “Toad” to “Prince.”

## 5 Discussion

Based on the analysis above, we can see that TT1 generally adopts literal translation, however, on some occasions the translation does not accurately convey the original meaning. In comparison, TT2 chooses more flexible translation approaches to convey the nuanced connotations of ST to the target readers, while also reflecting the translator’s interpretation of ST, such as addition of words, amplification of the information from the source text, provision of explanatory details, and moderate modifications to the original content.

**Figure 1:**
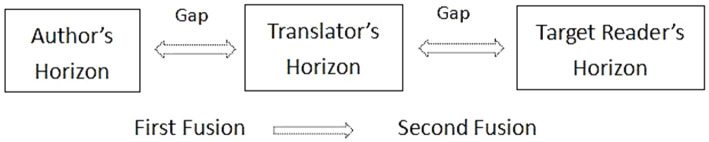
Two fusions of horizons in translation.

Zhu (2003) believes that there are two fusions of horizons happening during translation. The first fusion is between the translator’s horizon and the author’s horizon (or horizon of ST). Then there is a second fusion, between this fused horizon and the target reader’s horizon, as shown in Figure 1.

The gap that exists between different horizons is called the “horizon gap” (Zhu, 2003). For the translator, the first challenge is to bridge the horizon gap between the author/ST and themselves. The translator’s horizon refers to their knowledge, experience, understanding, ideology and attitudes, including not only the translator’s prejudices before translation, but also all the information gathered during the translation process (Zhu, 2003). In the dialogue with the text, the translator adjusts their individual pre-structure to the structure of the source text to achieve the fusion between the two fields of horizons (Li, 2012). Chen, the translator of TT2, is herself a psychotherapist. In an interview, she said:

The book is wrapped in the shell of a fairy tale, on the surface it appears to be a lovely pastoral fairy tale…However, its core aims at adults, concerning the transformation of adults, and how adults explore themselves spiritually. It encourages people to understand the psychological and personality factors that constitute themselves, and examine how the “me” of today came into being by looking back on their childhood (Yangzi Evening, 2022).

Based on her insight into the book, and being well aware of the theoretical framework of counselling, Chen made every effort to bring out the contextual and intended meaning of the key concepts of psychotherapy during translation.

The second challenge a translator faces is to bridge the horizon gap between themselves and the target reader. The target reader’s horizon is the translator’s imagination of their expectation of the translated text. To achieve the fusion between the translator and the target reader, “the translator must translate the meaning to be understood into the context in which the other speaker lives” (Gadamer, 2004, p. 386). The translator must state clearly how they understand, and every translation that takes its task seriously is at once clearer and flatter than the original (Gadamer, 2004).

Chen noticed that in the title of the original book, “Toads” is plural, suggesting that the author wanted to write this book for every “Mr. Toad” who has ever felt helpless. The target reader of her translation is supposed to be any person who needs psychological help. She tried to engage in a dialogue with her target reader. For her, translation is a second creation and a bilateral dialogue (Yangzi Evening, 2022). Chen went to great lengths to give the target reader, who lacks knowledge of psychotherapy, her own interpretations of the original text with reference to the whole theoretical framework of TA, hence forging the fusion of horizons between herself and the target reader.

In summary, psychotherapy guidebooks, unlike ordinary literary works, are usually embedded in a systematic theoretical framework of psychotherapy, which poses challenges for translation. To overcome these challenges, the translator must first endeavour to expand their horizon, that is, to accumulate psychotherapy knowledge both prior to and during translation. This expansion of knowledge mitigates the risk of prejudices leading to incorrect or insufficient understandings. Next, according to Gadamer’s Hermeneutical Circle, the meaning of a text is constructed through an ongoing conversation between the interpreter and the text, hence the translator should engage actively in a dialogue with the author/ST, comprehending the essence of pivotal terms and concepts by referring to the entire knowledge structure of psychotherapy, that is, “understand the whole in terms of the detail and the detail in terms of the whole.” It is through this iterative process that the first fusion of horizons between the translator and the author/ST can be facilitated. Finally, given that psychotherapy guidebooks are intended for general readers without an extensive background in this field, the translator should adopt innovative and flexible translation methods to help the reader overcome the horizon gap. According to Gadamer, every translation is at the same time an interpretation. The translator should strive to engage in a dialogue with the target reader and reconstruct the text as they perceive it, so that the second fusion of horizons between the translator and the target reader can be obtained.

## 6 Conclusion

To conclude, this study delves into the translation of psychotherapy guidebooks exemplified by the Chinese translation of *Counselling for Toads: A Psychological Adventure*, through the lens of Gadamer’s theory of philosophical hermeneutics. This study contributes to the field of translation studies and the practice of psychotherapy in the following two aspects. Firstly, it explores the translation of psychotherapy guidebooks through a detailed comparative case analysis of the two Chinese versions of the original book. Secondly, it proposes an appropriate strategy for the translation of psychotherapy guidebooks based on Gadamer’s theory of philosophical hermeneutics, to serve as a reference for future translators of this type of text. However, it is important to note that this study focuses on the translation of one psychotherapy guidebook, and it has limitations in evaluating the characteristics of other books of this type. Additionally, it mainly dwells on the translation of the key concepts of TA, so other probable problems of translating psychotherapy guidebooks are without consideration. Finally, this study is restricted to the methodology of qualitative case analysis, which may not be convincing enough. In the future, it is conceivable that more diverse research methods like interviews, questionnaires, and corpora could provide in-depth and more persuasive research in this field.

## Data Availability

The original contributions presented in the study are included in the article/supplementary material, further inquiries can be directed to the corresponding author.

## References

[ref1] Berne E. (1961). Transactional analysis in psychotherapy. New York: Grove Press, Inc.

[ref2] Berne E. (1975). What do you say after you say hello? London: Souvenir Press.

[ref3] Board R. D. (1997). Counselling for toads: A psychological adventure. London/ New York: Routledge.

[ref4] Board R. D. (2013). 蛤蟆先生的希望 (tr. M. H. Zhang). Nanjing: Yilin Press.

[ref5] Board R. D. (2020). 蛤蟆先生去看心理医生 (tr. Y. Chen). Tianjin: Tianjin People’s Press.

[ref6] Bradford D. T. Abilio M. (1993). Translation in bilingual psychotherapy. Prof. Psychol. Res. Pract. 24, 52–61. doi: 10.1037/0735-7028.24.1.52

[ref7] Corcodel S. Gabriela S. (2022). Clinical psychology terminology from a translation perspective. Retrieved May 30, 2023, from 10.5281/zenodo.6520455

[ref8] Dai W. J. Gu M. D. (2019). Misreading and enlightened Reading in English translations of key words in Wenxin Diaolong. Available online at: https://dspace.xmu.edu.cn/handle/2288/171260

[ref9] Ding L. F. (2012). A study on the cultural factors in the translation of ancient poetry in light of Gadamer’s hermeneutics. Yuwen J. 7, 64–66.

[ref10] Dong J. F. (2022). *A report on the translation of advances in contemplative psychotherapy: Accelerating healing and transformation (chapter 2).*MTI thesis.MTI thesis. Chongqing: Sichuan International Studies University.

[ref11] Gadamer H. G. (2004). Truth and method. London/New York: Continuum.

[ref12] Li Y. (2012). On the translator’s subjectivity--from the persepctive of Gadamer’s philosophical hermeneutics. High. Educ. Soc. Sci. 3, 21–26.

[ref13] Li J. X. (2021). *Analysis on the female images of D. H. Lawrence’s women in love and three translators’ interpretation from fusion of horizons*. MA thesis. Shanghai: Shanghai International Studies University.

[ref14] Li D. J. Sun J. H. (2021). Understanding and translation of Chinese classics: inspiration and reflection from Gadamer’s ontological hermeneutics. Foreign Lang. China 18, 101–109.

[ref15] Liu S. Y. Ren X. F. (2010). Applications of philosophical hermeneutics in literary translation process--comparative study between two Chinese versions of *moment in Peking*. J. Hebei Polytech. Univ. 10, 162–164.

[ref16] McGreevy J. Orrevall Y. Belqaid K. Bernhardson B. M. (2014). Reflections on the process of translation and cultural adaptation of aninstrument to investigate taste and smell changes in adults with cancer. Scand. J. Caring Sci. 28, 204–211. doi: 10.1111/scs.12026, PMID: 23383751

[ref17] Milne A. L. (2017). From cultural translation to clinical consultation: working between languages, working between discipline. Critic. Multiling. Stud. 5, 59–84.

[ref18] Paiano R. Teixeira M. C. T. V. Cantiere C. N. Efstratopoulou M. A. Carreiro L. R. R. (2019). Translation and cross-cultural adaptation of the motor behavior checklist (MBC) into Brazilian Portuguese. Trends Psychiatry Psychother. 41, 167–175. doi: 10.1590/2237-6089-2017-0104, PMID: 31166562

[ref19] Peng J. A. (2021). The translation strategies of culture-loaded verbs in Gladys’ translation of Bian Cheng from the perspective of the fusion of horizons. Beijing: Beijing Foreign Studies University.

[ref20] Rechtman R. (1997). Transcultural psychotherapy with Cambodian refugees in Paris. Transcult. Psychiatry 34, 359–375. doi: 10.1177/136346159703400305

[ref21] Ren C. L. (2021). A translation practice report on nurturing resilience: helping clients move forward from developmental trauma (chapter two). MTI thesis. Baotou: Inner Mongolia University of Technology.

[ref22] Rosa M. Metcalf E. Rocha T. B. M. Kieling C. (2018). Translation and cross-cultural adaptation into Brazilian Portuguese of the mood and feelings questionnaire (MFQ) – long version. Trends Psychiatry Psychother. 40, 72–78. doi: 10.1590/2237-6089-2017-0019, PMID: 29641647

[ref23] Sarvghadi F. Taebi N. Z. (2021). The translator-text interaction based on Gadamer’s theory of fusion of horizons: a case study of translations of romantic poetry into Persian. Hikma 20, 45–70. doi: 10.21071/hikma.v20i1.12787

[ref24] Stewart I. (1996). Developing transactional analysis counselling. London: Sage.

[ref25] Stewart I. Joines V. (1987). TA Today. Nottingham: Lifespace.

[ref26] Tan W. J. (2012). Taoist canon translation and Gadamer’s philosophical hermeneutics: With C. Spurgeon Medhurst’s translation of Tao Teh king as case of study. Chengdu: Southwest Jiaotong University.

[ref27] Tudor K. (Ed.) (2002). Transactional analysis approaches to brief therapy. London/ Thousand Oaks/ New Delhi: SAGE Publications Ltd.

[ref28] Wang J. (2020). A report on the translation of under pressure: Confronting the epidemic of stress and anxiety in girls (chapter one). Zhengzhou: Henan University.

[ref29] Weiss H. (2024). Some remarks on Freud as “translator” and translating Freud. Int. J. Psychoanal. 105, 746–756. doi: 10.1080/00207578.2024.2395739, PMID: 39576084

[ref30] West M. I. (2000). Counselling for toads: a psychological adventure (review). Lion Unicorn 24, 323–325. doi: 10.1353/uni.2000.0025

[ref31] Wu S. Yang X. Xia H. Li Y. He X. (2020). Discussion on Chinese ancient literature translation based on the English translation of the book of songs. Rev. Educ. Theory 3, 28–31. doi: 10.30564/ret.v3i2.1836

[ref32] Xu X. J. (2014). The analysis of different translations of the analects by James Legge and Ku hung-Ming from perspective of hermeneutics. Stud. Lit. Lang. 9, 149–154.

[ref33] Yan R. L. (2020). An analysis of Lin Wusun’s English translation of the analects from the perspective of fusion of horizons. Shenzhen: Shenzhen University.

[ref34] Yangzi Evening (2022). The self-awakening of the adult, with spice and bitterness-- an exclusive interview with Chen Ying, The translator of *counselling for toads: A psychological adventure*. Available online at: https://new.qq.com/rain/a/20220811A07TH900.html

[ref35] Zhong M. J. (2018). Hearing of divinity--an analysis of Holerlin’s poetry based on Gadamer’s hermeneutics. Chengdu: Southwest Jiaotong University.

[ref36] Zhu J. P. (2003). Inter-cultural interpretation in translation: A mode of philosophical hermeneutics and aesthetics of reception. Shanghai: East China Normal University.

